# Composition dependent effects of charged lipids on freeze-thaw induced fusion in dioleoyl phospholipid membranes

**DOI:** 10.2142/biophysico.bppb-v23.0026

**Published:** 2026-08-01

**Authors:** Gakushi Tsuji

**Affiliations:** 1 Department of Applied Chemistry and Biotechnology, Graduate School of Engineering, University of Fukui, Fukui 910-8507, Japan; 2 Life Science Innovation Center, University of Fukui, Fukui 910-8507, Japan

**Keywords:** Liposome, freeze-thaw, flow cytometry, artificial cell

## Abstract

Artificial cells based on giant unilamellar vesicles (GUVs) provide a platform for reconstituting biochemical reactions within membrane-bound compartments. However, limited membrane permeability restricts substrate exchange, leading to depletion during prolonged reactions. Freeze-thaw (F/T) treatment has been used to overcome this limitation by enabling liposome fusion and content exchange. Here, we systematically investigated F/T-induced fusion in dioleoylphosphatidylcholine (DOPC) liposomes with negatively charged dioleoylphosphatidylglycerol (DOPG) and positively charged dioleoyltrimethylammoniumpropane (DOTAP). DOPC liposomes exhibited internal content mixing comparable to POPC-based systems, whereas DOPG incorporation reduced encapsulation efficiency and did not enhance fusion, indicating that charge-mediated effects are not universal. In DOPC/DOTAP liposomes, dual-fluorescent populations detected by flow cytometry were largely attributed to membrane adsorption and aggregation rather than true internal content mixing, as confirmed by fluorescence microscopy. Although limited mixing occurred at low DOTAP fractions, higher DOTAP content promoted non-productive interactions. These results demonstrate that electrostatic interactions alone are insufficient to drive productive fusion and that fusion behavior depends strongly on lipid composition. In particular, hydrophobic acyl-chain structure, in addition to headgroup charge, plays a critical role in determining fusion efficiency and compartment stability.

## Significance

Freeze–thaw (F/T) fusion is widely used to induce content exchange in liposome-based artificial cells, yet its dependence on membrane composition remains poorly understood. Here, we show that the effects of charged lipids on F/T-induced fusion are not universal across phosphatidylcholine membranes. In DOPC-based systems, incorporation of negatively charged lipids does not enhance content mixing, in contrast to previous observations in POPC membranes. These findings demonstrate that electrostatic interactions alone are insufficient to drive productive fusion and highlight the importance of lipid packing and acyl-chain structure in governing membrane behavior under freezing conditions.

## Introduction

Lipid bilayer vesicles, or liposomes, can retain aqueous solutions within their internal compartments and encapsulate biomacromolecules such as proteins and substrates required for biochemical reactions [1]. Owing to these properties, liposomes have been widely used to encapsulate and reconstitute biochemical processes, including DNA and RNA self-replication [2,3], transcription [4,5], and translation [6], thereby serving as simplified models of cellular systems [7,8]. However, liposomes composed of phospholipid bilayers typically retain proteins and small molecules with minimal leakage after preparation, while simultaneously limiting the uptake of substrates from the external environment. To address this limitation, approaches that enable the exchange of internal contents through liposome-liposome fusion have been explored as efficient methods for supplying substrates to encapsulated reactions. Various fusion methods have been reported, including electrofusion [9,10], adhesion-mediated fusion driven by interactions between membrane proteins [11–13], pH-jump induced fusion [14], and freeze-thaw induced fusion [15,16]. Among these methods, freeze-thaw treatment enables repeated fusion cycles simply by increasing liposome density and modulating temperature. This approach has been used to repeatedly supply substrates for RNA replication and ribozyme reactions by employing rapid freezing [5,15–19]. In addition, slow-freezing protocols have been shown to concentrate substrates and thereby promote intraliposomal reactions [20]. Thus, freeze-thaw mediated fusion has been applied not only as a substrate delivery method in artificial cell models but also as a model system for studying growth and division processes in primitive cells that acquired phospholipid membranes [21].

In artificial cell research, POPC and DOPC phospholipids are commonly used to facilitate internal content mixing during freeze-thaw cycles [15,16]. These two phospholipids yield GUVs with similar morphologies when prepared using the water-in-oil emulsion transfer method [22,23], and both have been reported to support transcription and translation reactions [24–26]. However, whether the effects of charged lipid incorporation on freeze-thaw-induced fusion are generalizable across different phosphatidylcholine backbones remains unclear. In this study, we examined whether the properties we previously reported for POPC/POPG liposomes, a reduced rupture rate during freeze-thaw treatment and a higher proportion of liposomes exhibiting internal content mixing compared with POPC alone [17], are also observed for the different phospholipid pair DOPC/DOPG membrane. This comparison enables us to directly test the generality of charge-mediated effects in membrane fusion.

Furthermore, because fusion efficiency decreases in POPC/DOTAP liposomes during freeze-thaw treatment [17], we investigated whether this reduction arises from electrostatic interactions between oppositely charged membranes or from differences in membrane physical properties, such as acyl-chain composition and phase behavior. To disentangle these effects, we systematically analyzed DOPC/DOTAP liposomes before and after freeze-thaw treatment, and further examined fusion between negatively and positively charged liposomes. Our results reveal that, in contrast to POPC-based systems [17], incorporation of charged lipids into DOPC membranes does not enhance fusion efficiency and instead introduces limitations such as reduced encapsulation efficiency and non-productive membrane interactions. In particular, electrostatic attraction was found to promote aggregation and membrane adsorption rather than productive internal content mixing. These findings indicate that the effects of charged lipids on freeze-thaw-induced fusion are not universal and depend strongly on membrane composition. Together, our study highlights that hydrophobic acyl-chain structure, in addition to headgroup charge, plays a critical role in determining fusion behavior.

Taken together, these observations led us to investigate whether DOPC-based artificial cell systems are suitable for freeze-thaw-induced membrane interaction and internal content exchange. We aimed to determine how phospholipid composition influences membrane interaction behavior, compartment stability, and population-level fluorescence redistribution during freeze-thaw treatment, thereby assessing the suitability of DOPC-containing membranes for artificial cell systems utilizing freeze-thaw-mediated molecular exchange.

## Materials and methods

### Materials

1,2-Dioleoyl-sn-glycero-3-phosphocholine (DOPC), 1,2-Dioleoyl-sn-glycero-3-phosphoglycerol (DOPG), and 1,2-dioleoyl-3-trimethylammonium-propane (DOTAP) were purchased from Yuka-Sangyo Co., Ltd. (Tokyo, Japan). Transferrin Alexa Fluor 488 conjugate (TA488) and Transferrin Alexa Fluor 647 conjugate (TA647) were purchased from Life Technologies (Carlsbad, CA, USA). Mineral oil (heavy) was purchased from Wako (Osaka, Japan).

### Liposome preparation

Liposomes were prepared using a water-in-oil emulsion transfer method as previously described [23,26,27]. Briefly, DOPC, DOPG, and DOTAP were dissolved in chloroform (0.1 mg/μL) and mixed with mineral oil (heavy) to yield a final lipid concentration of 5 mg/mL. The lipid-oil mixture was heated at 80°C for 30 min. Lipids were combined at the desired molar ratios prior to emulsification.

The internal solution (50 mM HEPES-KOH, pH 7.6, 350 mM potassium glutamate, 500 mM sucrose) containing either transferrin-Alexa 488 (TA488) or transferrin-Alexa 647 (TA647) at 1.5 μM was added to the lipid-oil mixture and emulsified. The emulsion was incubated on ice for 10 min and then gently layered onto the outer solution (50 mM HEPES-KOH, 350 mM potassium glutamate, and 1 M glucose). Samples were centrifuged at 18,000×g for 30 min at 4°C. After centrifugation, the liposome pellet was collected through a hole at the bottom of the tube using a needle. The external solution was then replaced with incubation buffer (50 mM HEPES-KOH and 1 M glucose).

### Freezing-thawing treatment

Liposome fusion by freeze-thaw (F/T) treatment was performed as previously described [15,16]. Liposome suspensions containing equal numbers of liposome particles were mixed and centrifuged at 18,000×g for 10 min at 4°C.The resulting pellet was frozen in liquid nitrogen for 5 min and subsequently thawed at room temperature for 10 min. After thawing, samples were gently resuspended and centrifuged again at 18,000×g for 5 min at 4°C. The supernatant was then replaced with an equal volume of incubation buffer.

### Flow cytometry

Each liposome sample was analyzed by flow cytometry (FCM) using a FACS Aria II (BD Biosciences, San Jose, CA, USA). Forward scatter (FSC) and side scatter (SSC) signals were acquired to detect liposome populations. All comparative flow cytometry analyses within each experiment were performed using samples measured under identical acquisition conditions, instrument settings, and gating criteria during the same experimental session to minimize inter-experimental variability. Acquisition thresholds for both FSC and SSC signals were fixed at 200 throughout all flow cytometry measurements to reduce the detection of small debris and background particles prior to downstream gating analysis. Flow cytometry measurements were continued until sufficient GUV-gated populations were obtained for downstream analyses. Depending on the experimental condition, more than 10,000 GUV-gated events were analyzed per sample for fluorescence-based population analyses.

Flow cytometry data were analyzed in R using the prada package. Events corresponding to GUV populations were selected on FSC-A versus SSC-A plots using a predefined gate (SSC-A≥10^2.2^ and FSC-A≥0.025×(SSC-A)^1.8^) to exclude debris and non-vesicular particles [26,28]. The FSC/SSC gating strategy was determined based on previous artificial cell flow cytometry studies and was consistently applied throughout the experiments. The GUV ratio was calculated as the fraction of events within this gate relative to the total number of detected events. FSC-based apparent size distributions were evaluated from all gated events, and log_10_(FSC-A) values from three independent experiments were plotted as density distributions before and after freeze-thaw treatment.

Fluorescence from TA488 was excited with a 488 nm semiconductor laser and detected using a 530±15 nm band-pass filter (FITC-A). Fluorescence from TA647 was excited with a 633 nm HeNe laser and detected using a 660±10 nm band-pass filter (APC-A). The gated populations were additionally supported by fluorescence microscopy observations of micron-sized vesicular structures under the corresponding experimental conditions.

For inner content mixing assays, fluorescence intensities of TA488 and TA647 were measured for each liposome. Fluorescence-positive gates were determined based on the fluorescence distributions of single-labeled control GUVs and were consistently applied throughout each experimental session. Non-fluorescent blank liposomes were not independently analyzed in this study. Liposomes exhibiting fluorescence intensities above both thresholds were classified as double-positive liposomes, and the fraction of double-positive liposomes was used as a measure of inner content mixing.

### Fluorescence microscopy

Fluorescence images of liposomes were acquired using an Axio Observer 7 microscope equipped with a 63× Plan-Neofluar oil-immersion objective lens (NA=1.4) (Carl Zeiss, Oberkochen, Germany). Transferrin-Alexa 488 (TA488) fluorescence was excited using a 470 nm LED and detected using a 525±25 nm band-pass filter. Transferrin-Alexa 647 (TA647) was excited using a 590 nm LED and detected using a 629±31 nm band-pass filter. Fluorescence microscopy was used to qualitatively assess liposome morphology and the localization of fluorescent inner content signals.

### Statistics

Statistical analyses were performed using two-way analysis of variance (ANOVA), with membrane composition and freeze-thaw (F/T) treatment as independent factors, followed by Tukey’s post hoc multiple-comparison test. Data were obtained from three independent experiments (n=3) and are presented as mean±standard deviation (SD). Differences were considered statistically significant at p<0.05. All statistical analyses were performed using R version 3.6.3 (R Foundation for Statistical Computing, Vienna, Austria). Two-way ANOVA and Tukey’s post hoc tests were conducted using the stats package.

## Results

### Freezing-thawing induced liposome fusion composed of negatively charged phospholipids

First, we examined whether the incorporation of DOPG affected the stability of GUVs during freeze-thaw (F/T) treatment. Negatively charged lipids such as POPG have been reported to promote GUV formation in POPC-based systems [5,17]. In contrast, liposomes composed solely of DOPC exhibited a relatively high GUV yield (~60%) under our experimental conditions (Fig. 1A, B, 0% Before F/T), exceeding previously reported values for POPC systems [29]. However, increasing the fraction of DOPG resulted in a progressive decrease in GUV yield prior to F/T treatment (Fig. 1A, B, Before F/T).

Following a single F/T treatment using liquid nitrogen, the GUV yield decreased in DOPC and DOPC/DOPG (10%) membranes, whereas little change was observed at ≥30% DOPG (Fig. 1A, B, After F/T). Two-way ANOVA revealed significant effects of both lipid composition (p<0.001) and F/T treatment (p<0.01) on the fraction of GUVs, as well as a significant interaction between the two factors (p<0.05). Tukey’s post hoc test further showed that F/T treatment significantly reduced the GUV fraction in DOPC and DOPG10 liposomes (Tukey-adjusted p<0.05), whereas no significant differences were observed in DOPG30 or DOPG50 liposomes. These results indicate that the effects of F/T treatment on GUV populations depended on membrane composition. Differential interference contrast (DIC) images revealed that overall vesicle morphology remained largely spherical across all conditions, although a reduction in apparent vesicle size after F/T treatment was observed (Fig. 1C). In addition, DOPG-containing samples exhibited increased lipid aggregation, consistent with the FCM observations.

To further evaluate size changes, FSC-based density distributions were constructed from gated GUV populations (Fig. S1). For DOPC and DOPG 10%, the distributions shifted toward lower FSC values after F/T, indicating a reduction in apparent vesicle size. In contrast, little change in the FSC distribution was observed for membranes containing ≥30% DOPG. Notably, the fraction of large vesicles (high FSC region) was already low prior to F/T under these conditions, suggesting that the apparent stability does not necessarily reflect reduced rupture during F/T but rather the initial size distribution and scatter heterogeneity of the GUV population.

Next, we analyzed the fluorescence intensities of transferrin-Alexa 488 (TA488) and transferrin-Alexa 647 (TA647) encapsulated within liposomes to evaluate internal content mixing (Fig. 2A). The analyses presented in Fig. 2 were performed using the same liposome preparations and experimental conditions analyzed in Fig. 1. Before F/T treatment, liposomes containing either fluorescent protein exhibited distinct fluorescence populations in FCM analysis. However, the encapsulation efficiency decreased with increasing DOPG content (Fig. 2B, Before F/T; Fig. S2A), in contrast to previously reported POPC/POPG systems [5,17]. Fluorescence microscopy confirmed that the fluorescent proteins were localized within the lumen of liposomes, and no significant membrane-associated fluorescence was observed even at higher DOPG fractions (Fig. S2B).

After F/T treatment, double-positive populations were detected across all lipid compositions. In DOPC and 10% DOPC/DOPG liposomes, internal content mixing was clearly detected by both FCM and fluorescence microscopy (Fig. 2B, C). In liposomes containing ≥30% DOPG, double-positive populations were still detected, however, the extent of mixing was reduced compared with that in low-DOPG conditions (Fig. 2D). Two-way ANOVA revealed significant effects of both lipid composition (p<0.001) and F/T treatment (p<0.001) on the fraction of double-positive liposomes, together with a significant interaction between the two factors (p<0.01). Tukey’s post hoc test showed that F/T treatment significantly increased the fraction of double-positive liposomes in DOPC and 10% DOPG liposomes (p<0.01), whereas no significant changes were observed in DOPG30 or DOPG50 liposomes. Furthermore, after F/T treatment, the fractions of double-positive liposomes in 30% and 50% DOPG membranes were significantly lower than those in DOPC and 10% DOPG membranes (p<0.05).

Notably, in contrast to POPC-based systems [17], where incorporation of ~10% POPG enhances internal content mixing efficiency, no such enhancement was observed in DOPC/DOPG liposomes (Fig. 2D). Instead, increasing DOPG content resulted in a gradual decrease in mixing efficiency. These results indicate that the effect of negatively charged lipids on F/T-induced content mixing is not universal across phosphatidylcholine membranes and is strongly dependent on the acyl-chain composition.

### Freezing-thawing induced liposome fusion composed of positively charged phospholipids

Next, we analyzed internal content mixing in liposomes composed of DOPC and the positively charged lipid DOTAP following a single freeze-thaw (F/T) treatment. In contrast to DOPG-containing liposomes, incorporation of DOTAP did not reduce the GUV population. Instead, a relatively high fraction of GUVs was maintained across all compositions (Fig. 3A; Fig. S3A). DIC microscopy image confirmed the presence of intact, spherical GUVs prior to F/T treatment (Fig. S3B). FSC-based size distributions before and after F/T revealed a slight shift toward smaller sizes at 50% DOTAP, whereas no clear peak shift was observed under other conditions (Fig. S3C). Two-way ANOVA revealed significant effects of lipid composition (p<0.05) and F/T treatment (p<0.01), together with a significant interaction between the two factors (p<0.05). Tukey’s post hoc test revealed no significant differences before and after F/T treatment in DOTAP-containing liposomes.

Consistent with previous reports using POPC/DOTAP systems [17], particles exhibiting both fluorescence signals were detected by FCM even before F/T when liposomes encapsulating different fluorescent proteins were mixed (Fig. S4A, Before F/T). This dual fluorescence signal was not due to internal content mixing, as confirmed by microscopy, but was instead attributed to membrane-associated fluorescence or aggregation of liposomes (Fig. S4B). Accordingly, GUVs exhibiting true internal content mixing were not observed in the before F/T samples.

The fluorescence intensity distribution further differed from that observed in DOPC/DOPG liposomes. At 10% DOTAP, the proportion of GUVs exhibiting high fluorescence intensity decreased, whereas this population increased at higher DOTAP fractions (30% and 50%) (Fig. S4C). However, even at 50% DOTAP, a substantial fraction of low-fluorescence GUVs remained, indicating that overall encapsulation efficiency was limited.

After F/T treatment, the proportion of liposomes exhibiting dual fluorescence increased according to FCM analysis and was higher in liposomes containing 30% and 50% DOTAP than in DOPC and 10% DOTAP liposomes (Fig. 3B). Two-way ANOVA revealed significant effects of both lipid composition (p<0.001) and F/T treatment (p<0.001) on the fraction of double-positive liposomes, whereas no significant interaction between the two factors was detected. Tukey’s post hoc test showed that F/T treatment significantly increased the fraction of double-positive liposomes in all membrane compositions examined (p<0.01). In addition, the fractions of double-positive liposomes in 30% and 50% DOTAP membranes were significantly higher than those in DOPC membranes after F/T treatment (p<0.001). However, fluorescence microscopy revealed substantial heterogeneity within these populations, particularly at higher DOTAP fractions (Fig. S4B). Because aggregation-associated interactions observed in DOTAP-containing liposomes could potentially influence the proportion of double-positive populations calculated from total particle counts, we additionally evaluated the fraction of double-positive GUVs within the gated GUV population. Similar overall trends were observed in the GUV-gated analysis (Fig. S4D), indicating that the population-level fluorescence redistribution observed after F/T treatment was not solely attributable to changes in total particle populations. While liposomes containing 10% DOTAP exhibited internal content mixing, those containing ≥30% DOTAP predominantly showed fluorescence localized to the membrane rather than within the lumen (Fig. 3C). These observations suggest that the apparent increase in dual-fluorescent populations detected by FCM at higher DOTAP contents may partially reflect membrane-associated fluorescence interaction and/or aggregation-associated colocalization in addition to fusion-related events. To examine whether these dual-fluorescent populations reflected false-positive signals caused by nonspecific fluorescence increases in both channels induced by DOTAP-containing membranes, we additionally analyzed single-labeled liposomes containing either TA488 or TA647 before and after F/T treatment. In these control experiments, the single-labeled liposomes predominantly retained their original fluorescence distributions and did not produce the broad diagonal dual-positive populations observed in mixed-liposome samples (Fig. S5). These results suggest that the dual-positive populations observed in mixed-liposome samples cannot be explained solely by nonspecific fluorescence shifts induced by DOTAP-containing membranes.

These observations indicate that the apparent increase in dual-fluorescent liposomes detected by FCM at higher DOTAP contents primarily reflects membrane adsorption of leaked fluorescent proteins or aggregation, rather than true internal content mixing. A similar phenomenon has been reported in POPC/DOTAP systems [17], where fluorescent proteins leak during F/T and subsequently associate with the membrane. Therefore, high DOTAP fractions appear to complicate the interpretation of FCM-based internal content mixing analyses due to strong membrane-associated interactions and heterogeneous vesicle states.

### Freezing-thawing induced liposome fusion between negatively charged liposomes and positively charged liposomes

Next, we investigated fusion between negatively charged (DOPC/DOPG) and positively charged (DOPC/DOTAP) liposomes. At 50% charged lipid content, reduced protein encapsulation efficiency and pronounced membrane-associated fluorescence were observed. Therefore, subsequent experiments were performed using 10% and 30% of each charged lipid (Fig. 4A).

Before F/T, the GUV yield was lower in liposomes containing DOPG/DOTAP than in DOPC liposomes, indicating that the introduction of charged lipids reduced GUV formation prior to F/T treatment (Fig. 4B, Fig. S6A). Two-way ANOVA revealed a significant effect of lipid composition on the fraction of GUVs (p<0.001), together with a significant interaction between lipid composition and F/T treatment (p<0.001), whereas the main effect of F/T treatment was not significant. Tukey’s post hoc test showed that F/T treatment significantly reduced the GUV fraction in DOPC liposomes (Tukey-adjusted p<0.01), whereas no significant changes were observed in liposomes containing 10% or 30% DOPG/DOTAP. Furthermore, no significant differences in GUV fraction were detected among DOPC, 10%, and 30% DOPG/DOTAP liposomes after F/T treatment according to Tukey’s post hoc test. DIC microscopy confirmed the presence of GUVs under all conditions. However, pronounced aggregation was observed in samples containing 30% of each charged lipid (Fig. S6B, Before F/T). After F/T, GUVs were still observed, consistent with FCM measurements (Fig. S6A, B).

FCM analysis detected particles exhibiting both fluorescence signals even before F/T, particularly under 10% conditions, with a higher proportion at 30% (Fig. 4C, Fig. S7A). Two-way ANOVA revealed significant effects of both membrane composition (p<0.001) and F/T treatment (p<0.01) on the fraction of dual-positive populations, together with a significant interaction between the two factors (p<0.001), indicating that the effects of F/T treatment differed depending on the membrane compositions of oppositely charged liposomes. Tukey’s post hoc test revealed no significant changes in the fractions of dual-positive populations before and after F/T treatment in liposomes containing 10% or 30% DOPG/DOTAP. In addition, before F/T treatment, the fractions of dual-positive populations in 10% and 30% DOPG/DOTAP liposomes were significantly higher than those in DOPC liposomes (p<0.05). Fluorescence microscopy revealed that these pre-existing dual-fluorescent populations did not correspond to internal content mixing. Instead, they were associated with aggregated liposomes in which both fluorescent signals were colocalized (Fig. S7B). Nevertheless, after F/T treatment, fluorescence microscopy confirmed the presence of GUVs exhibiting internal content mixing under all conditions (Fig. 4D).

These results indicate that electrostatic attraction between oppositely charged liposomes promotes aggregation but does not necessarily lead to efficient internal content mixing. Instead, productive fusion appears to occur under moderate charge conditions, whereas excessive charge promotes non-productive interactions.

## Discussion

In this study, we systematically examined how membrane charge and lipid composition influence F/T induced fusion and internal content mixing in GUVs, focusing on DOPC based membranes in comparison with previously reported POPC-based systems [17].

We first found that DOPC-only liposomes exhibited a relatively high GUV yield prior to F/T (Fig. 1), comparable to or higher than that reported for POPC systems [29]. However, incorporation of the negatively charged lipid DOPG into DOPC membranes markedly reduced GUV yield even before F/T and did not improve internal content mixing after F/T. This reduction in GUV yield suggests that the number of vesicles capable of encapsulating fluorescent tracers was markedly decreased, which in turn limits the observable internal content mixing after F/T (Fig. 2). This contrasts with POPC/POPG systems, in which POPG enhances mixing efficiency while reducing leakage [17]. These results demonstrate that the effects of negatively charged lipids on F/T-induced fusion are not universal across phosphatidylcholine membranes. To further examine the role of electrostatic interactions, we analyzed DOPC/DOTAP liposomes. Although flow cytometry detected a substantial population of dual-fluorescent particles prior to F/T, fluorescence microscopy revealed that these signals originated primarily from membrane-associated adsorption or aggregation rather than internal content mixing. After F/T, internal content mixing was observed; however, at higher DOTAP fractions, a significant portion of dual-fluorescent particles still reflected non-productive interactions. Furthermore, when oppositely charged liposomes were combined, electrostatic attraction promoted aggregation but did not enhance mixing efficiency. These observations indicate that electrostatic interactions alone are insufficient to drive productive fusion accompanied by internal content exchange.

Together, these findings highlight that lipid composition governs fusion, rupture, and content mixing behavior during F/T treatment through mechanisms beyond simple charge effects. A key implication of this study is that differences in hydrophobic acyl-chain structure, such as symmetric unsaturation in DOPC versus asymmetric saturation in POPC, likely influence membrane fluidity, membrane packing, defect formation, and bilayer reconstitution during freezing and thawing. In particular, the lower membrane ordering and increased fluidity of dioleoyl lipid membranes may favor membrane deformation and aggregation-associated interactions rather than productive internal content mixing. In addition, acyl-chain symmetry may influence membrane mechanical properties relevant to membrane rupture, defect formation, and bilayer reconstitution during freezing and thawing. The symmetric dioleoyl chains of DOPC-based membranes (18:1/18:1) and the asymmetric palmitoyl-oleoyl chains of POPC-based membranes (16:0/18:1) are expected to differ in membrane packing, curvature elasticity, and membrane ordering. Such differences may influence how membranes deform, rupture, and reassemble during rapid freezing and thawing, thereby affecting whether membrane interactions result in productive internal content mixing or non-productive aggregation-associated interactions.

Because freeze-thaw-induced membrane remodeling is highly sensitive to membrane physical properties, the use of dioleoyl lipids in this study enabled us to evaluate charge-dependent membrane interactions under highly fluid membrane conditions. Our results suggest that such physical properties may determine whether membrane interactions lead to productive internal content mixing or non-productive aggregation-associated interactions. Although membrane fusion is often discussed in terms of highly curved intermediates such as stalk structures, the present liquid nitrogen-based F/T system likely involves substantial membrane disruption and reconstitution processes [18]. Therefore, differences in membrane physical properties associated with acyl-chain structure may strongly influence the balance between membrane rupture, leakage, aggregation, and internal content exchange during F/T treatment.

In the context of artificial cell systems, these results suggest that successful implementation of F/T mediated substrate exchange requires careful selection of membrane composition, considering both headgroup charge and acyl-chain properties. More broadly, the present findings demonstrate that membrane composition can function as a controllable design parameter governing compartment stability, membrane interaction states, aggregation behavior, and internal content exchange under external physical perturbations. Our findings provide practical guidelines for designing membrane compositions that enable efficient and reliable content exchange in liposome based artificial cells and also highlighting how specific lipid species can be used to modulate the physical and functional behaviors of synthetic cell systems.

## Conclusion

In this study, we systematically investigated F/T induced fusion and content mixing in DOPC based GUVs. We found that the incorporation of charged lipids, including both negatively and positively charged components, did not significantly enhance productive fusion accompanied by internal content mixing. In particular, electrostatic interactions promoted aggregation but were insufficient to drive efficient internal content exchange. In the case of negatively charged lipids, the marked reduction in encapsulation efficiency further limited the observable content mixing after F/T.

These results indicate that membrane properties beyond headgroup charge, such as lipid packing and acyl-chain structure, play a central role in determining fusion outcomes during F/T treatment. Notably, this behavior differs from previously reported systems using POPC and POPG [5,17], suggesting that the effects of charged lipids on F/T-induced fusion are not universal across phosphatidylcholine membranes.

Overall, our findings provide a framework for understanding how membrane composition governs the balance between fusion, leakage, and aggregation under freezing conditions, with implications for the design of liposome based artificial cells.

## Conflict of interest

The authors declare no conflicts of interest.

## Author contributions

G.T. designed the experiments, performed and analyzed the experimental data; and wrote the manuscript.

## Data availability

The evidence data generated and/or analyzed during the current study are available from the corresponding author on reasonable request.

## Figures and Tables

**Figure 1:**
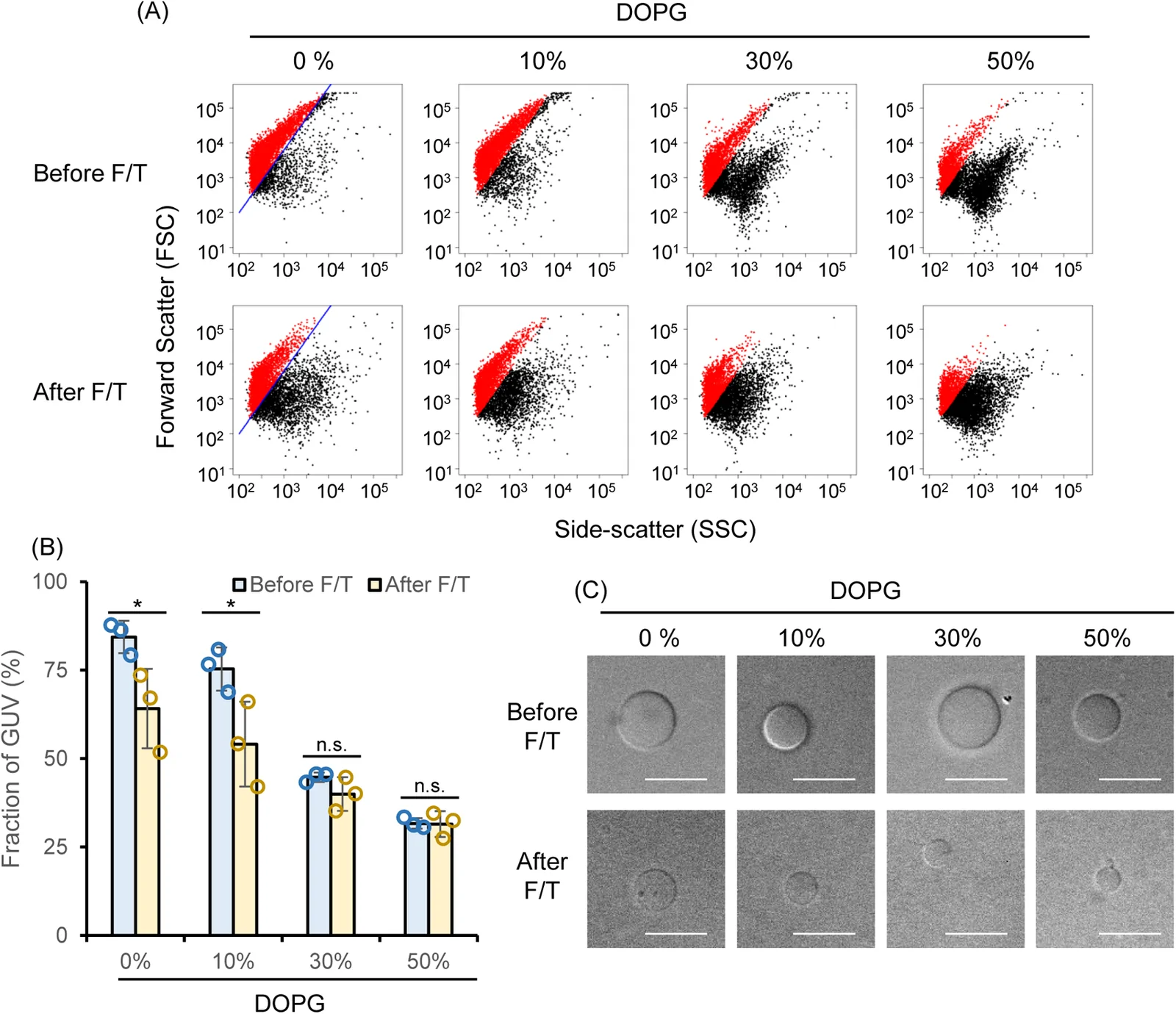
Light scattering and morphological analysis of GUVs prepared from DOPC and DOPG (A) Light-scattering analysis of liposomes measured by flow cytometry. Representative FSC–SSC dot plots of DOPC GUVs before and after freeze-thaw treatment are shown with the GUV gate indicated. The GUV gate was defined based on FSC–SSC profiles to include vesicle-sized events while excluding debris and non-vesicular particles. The same gating criteria were applied across all samples, and events within the gate are shown in red. (B) Average fraction of GUVs obtained from three independent experiments. Error bars represent the standard deviation (SD) of three independent experiments (n=3). Individual data points corresponding to each independent experiment are overlaid on the bar graphs. Statistical significance for comparisons before and after F/T treatment is indicated as *p<0.05 and n.s. (not significant), based on Tukey’s post hoc test. (C) Differential interference contrast microscopy images of liposomes composed of DOPC with or without DOPG. Scale bars, 10 μm.

**Figure 2:**
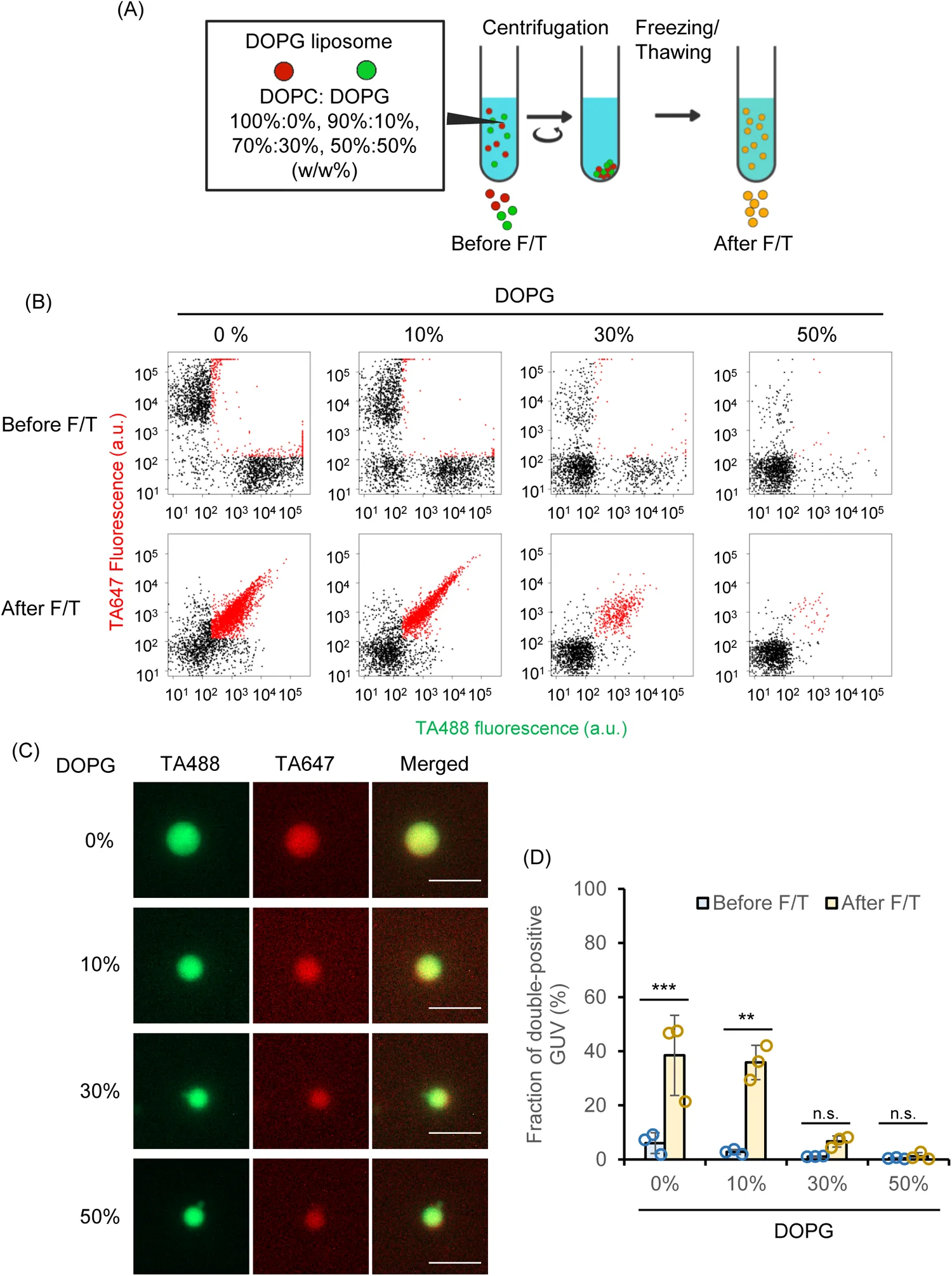
Freeze-thaw induced internal content mixing of GUVs prepared from DOPC and DOPG. (A) Schematic illustration of the experimental conditions. (B) Fluorescence of TA488 and TA647 measured by flow cytometry. Red dots indicate liposomes exhibiting fluorescence intensities of both TA488 and TA647 above the background level. (C) Fluorescence microscopy images of liposomes containing TA488 (green) or TA647 (red) after freeze-thaw treatment. Scale bar, 10 μm. (D) Average fraction of double-positive liposomes, calculated as the number of double-positive GUVs divided by the total number of detected particles. Error bars represent the SD of three independent experiments (n=3). Individual data points corresponding to each independent experiment are overlaid on the bar graphs. Statistical significance for comparisons between before and after F/T treatment is indicated as **p<0.01, ***p<0.001, and n.s. (not significant), based on Tukey’s post hoc test.

**Figure 3:**
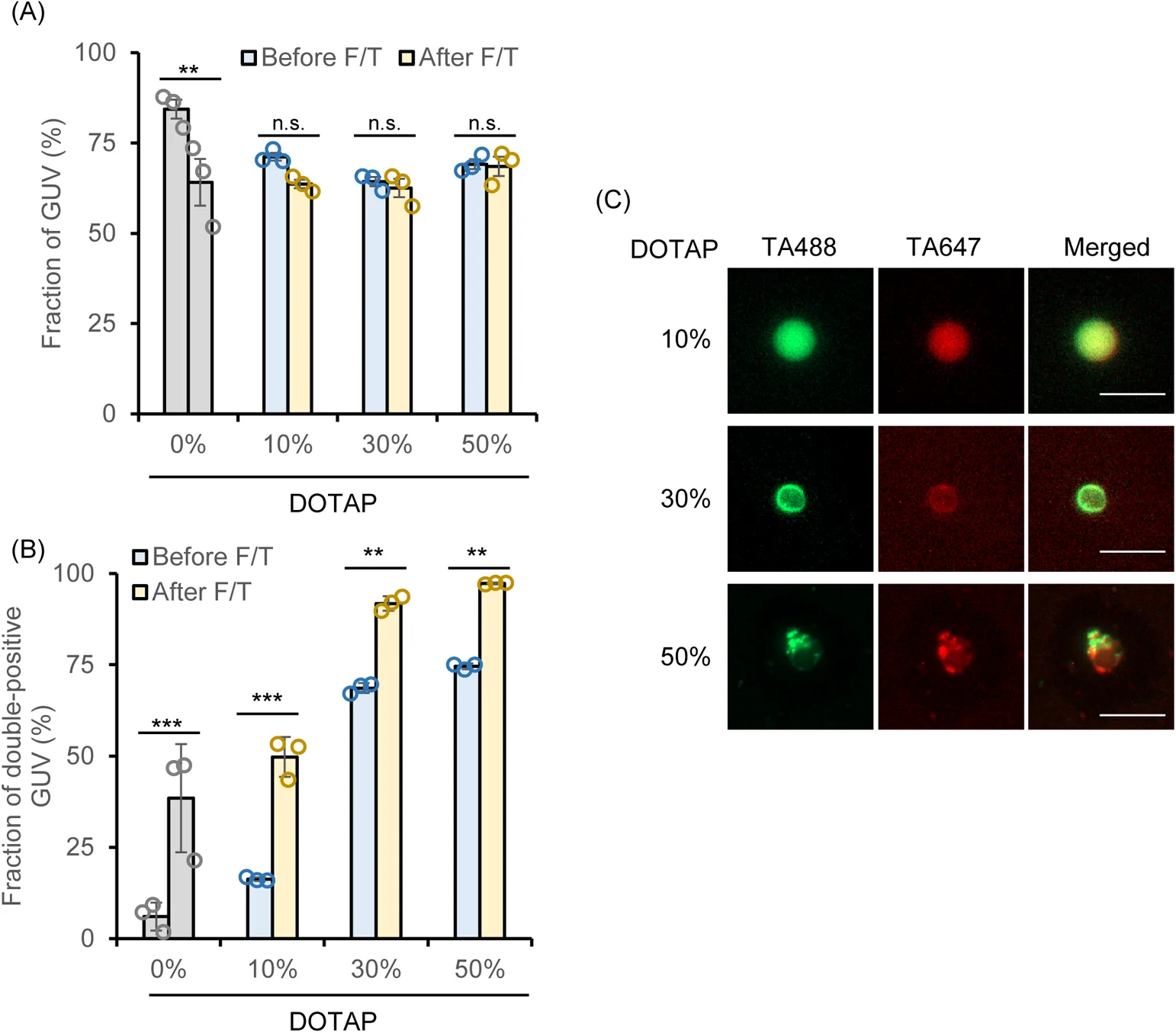
Freeze-thaw induced internal content mixing of GUVs prepared from DOPC and DOTAP. (A) Average fraction of GUVs obtained from three independent experiments. Error bars represent the SD of three independent experiments (n=3). Individual data points corresponding to each independent experiment are overlaid on the bar graphs. The 0% condition corresponds to the same DOPC control dataset presented in Fig. 1 is shown in gray to indicate the shared control condition. Statistical significance for comparisons between before and after F/T treatment is indicated as **p<0.01 and n.s. (not significant), based on Tukey’s post hoc test. (B) Average fraction of double-positive liposomes, calculated as the number of double-positive GUVs divided by the total number of detected particles. Error bars represent the SD of three independent experiments (n=3). Individual data points corresponding to each independent experiment are overlaid on the bar graphs. The 0% condition corresponds to the same DOPC control dataset presented in Fig. 2 is shown in gray to indicate the shared control condition. Statistical significance for comparisons between before and after F/T treatment is indicated as **p<0.01 and ***p<0.001, based on Tukey’s post hoc test. (C) Fluorescence microscopy images of liposomes containing TA488 (green) or TA647 (red) after freeze-thaw treatment. Scale bar, 10 μm.

**Figure 4:**
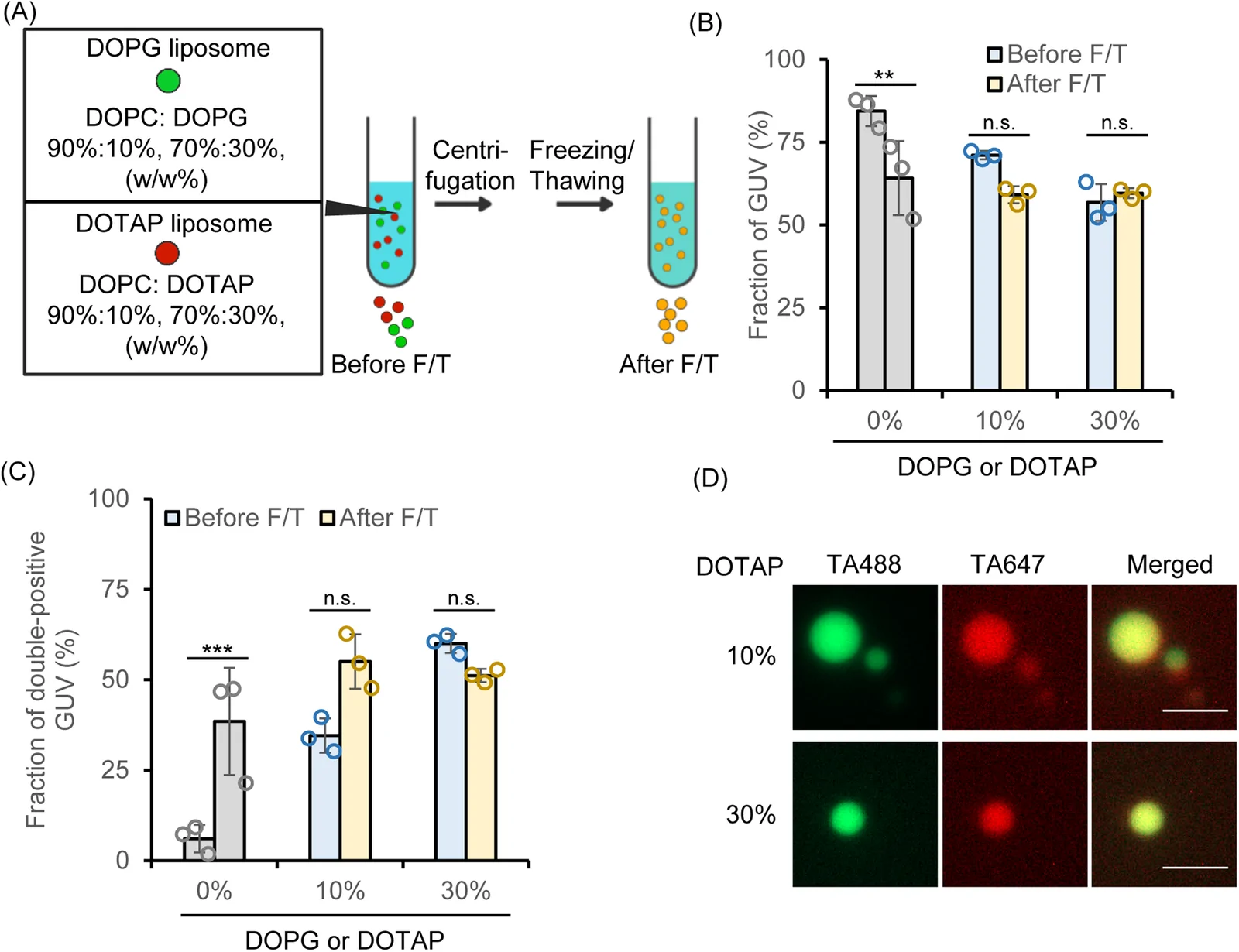
Freeze-thaw induced internal content mixing between DOPG-containing GUVs and DOTAP-containing GUVs. (A) Schematic illustration of the experimental conditions. (B) Average fraction of GUVs obtained from three independent experiments. Error bars represent the SD of three independent experiments (n=3). Individual data points corresponding to each independent experiment are overlaid on the bar graphs. The 0% condition corresponds to the same DOPC control dataset presented in Fig. 1 is shown in gray to indicate the shared control condition. Statistical significance for comparisons between before and after F/T treatment is indicated as **p<0.01 and n.s. (not significant), based on Tukey’s post hoc test. (C) Average fraction of double-positive liposomes, calculated as the number of double-positive GUVs divided by the total number of detected particles. Error bars represent the SD of three independent experiments (n=3). Individual data points corresponding to each independent experiment are overlaid on the bar graphs. The 0% condition corresponds to the same DOPC control dataset presented in Fig. 2 is shown in gray to indicate the shared control condition. Statistical significance for comparisons between before and after F/T treatment is indicated as ***p<0.001 and n.s. (not significant), based on Tukey’s post hoc test. (D) Fluorescence microscopy images of liposomes containing TA488 (green) or TA647 (red) after freeze-thaw treatment. Scale bar, 10 μm.
