# Supplementary material for: Composition dependent effects of charged lipids on freeze-thaw induced fusion in dioleoyl phospholipid membranes
Source: Biophys Physicobiol. 2026 Aug 1;23(3):e230026. doi: 10.2142/biophysico.bppb-v23.0026 (PMC13639342; doi:10.2142/biophysico.bppb-v23.0026)
Supplement: Supplementary file 1 [file 23_e230026_1.pdf]

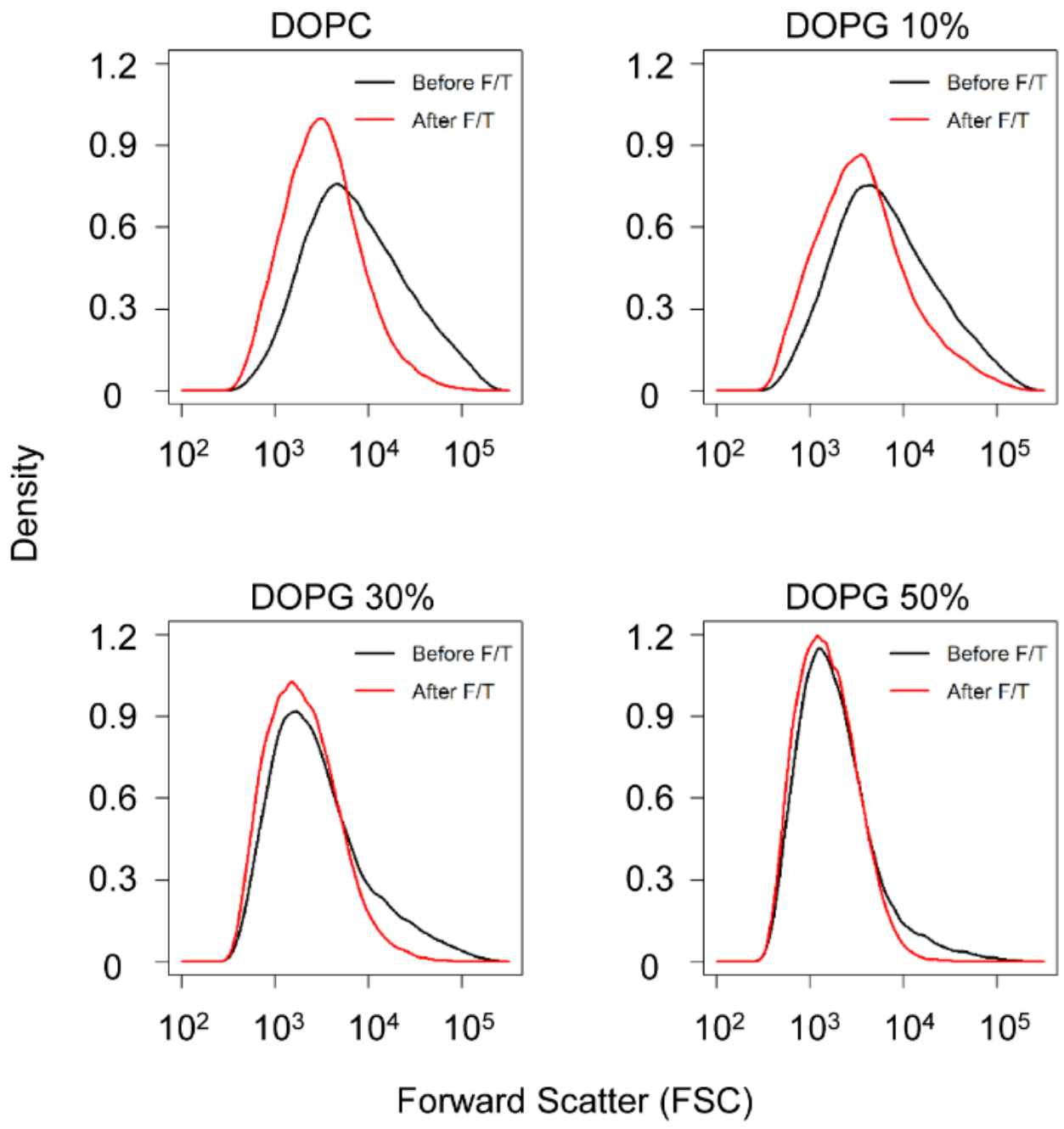

Supplementary Figure S1. FSC-based relative size distributions of GUVs before and after freeze-thaw treatment. Flow cytometry events within the predefined GUV gate were used to evaluate FSC-based apparent size distributions. For each membrane composition, $\log_{10}$(FSC-A) values from three independent experiments were pooled and plotted as density curves. Black lines indicate samples before freeze-thaw treatment, and red lines indicate samples after F/T treatment.

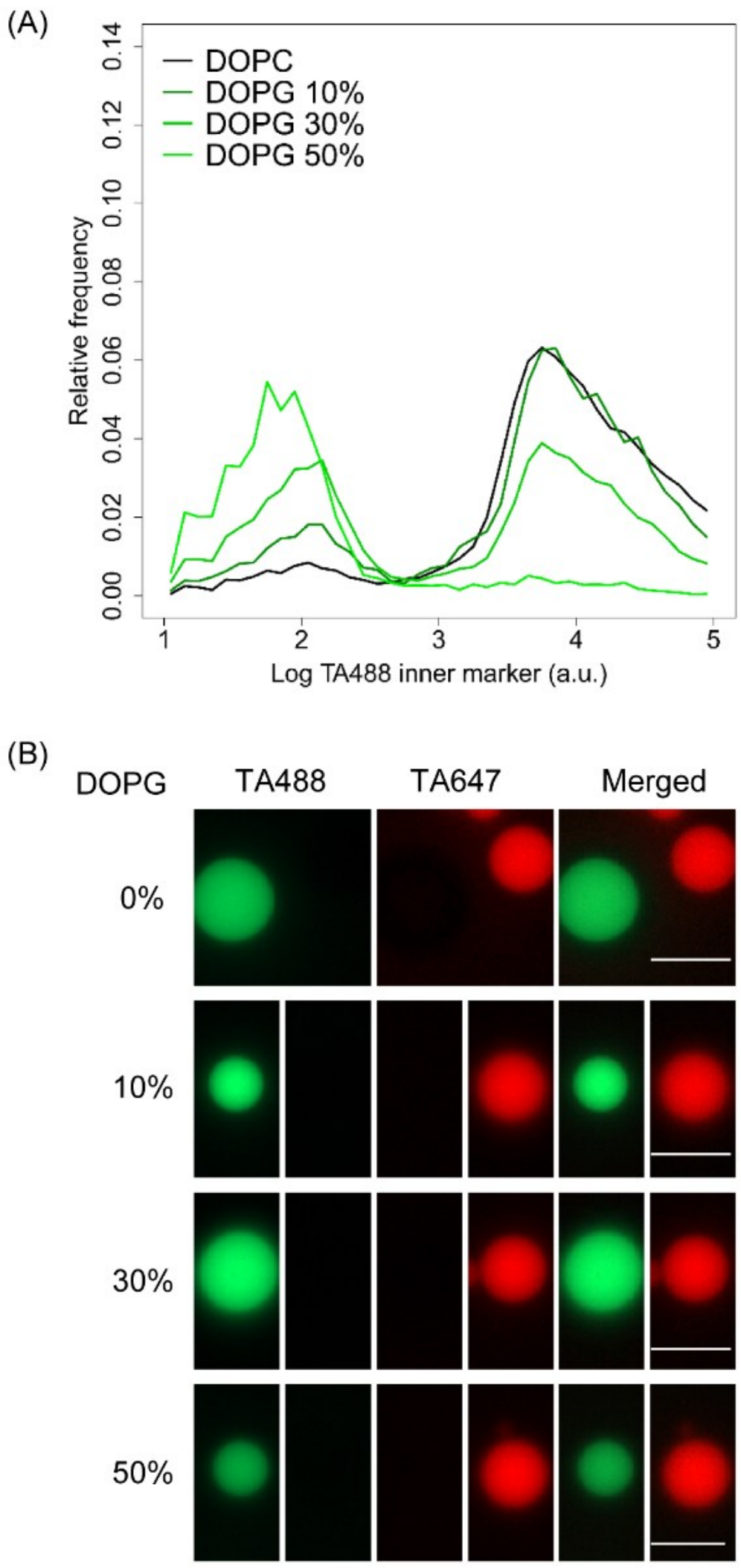

Supplementary Figure S2. Encapsulation efficiency and fluorescence microscopy of GUVs prepared from DOPC and DOPG**.** (A) Histogram of TA488 fluorescence intensity inside GUVs before freeze-thaw treatment at various DOPG concentrations. The vertical axis represents the relative frequency. (B) Fluorescence microscopy images of liposomes containing TA488 (green) or TA647 (red) before freeze-thaw treatment. Scale bar, 10 μm.

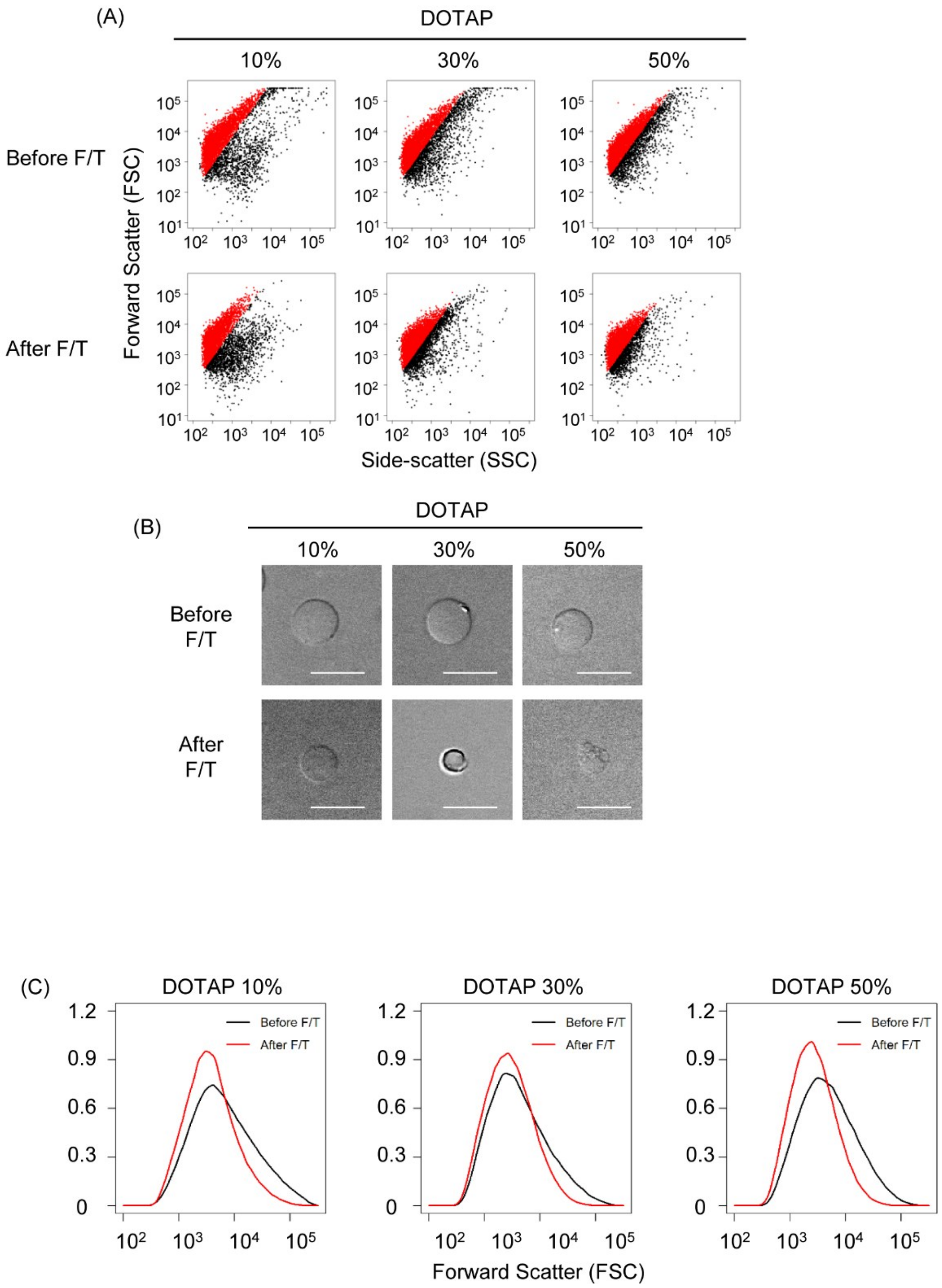

Supplementary Figure S3. Light scattering and morphological analysis of GUVs prepared from DOPC and DOTAP. (A) Light-scattering analysis of liposomes measured by flow cytometry. Red dots indicate GUVs. (B) Differential interference contrast microscopy images of liposomes composed of DOPC with or without DOTAP. Scale bars, 10 μm. (C) Flow cytometry analysis within the predefined GUV gate were used to evaluate FSC-based apparent size distributions. For each membrane composition, $\log_{10}$(FSC-A) values from three independent experiments were pooled and plotted as density curves. Black lines indicate samples before freeze-thaw treatment, and red lines indicate samples after F/T treatment.

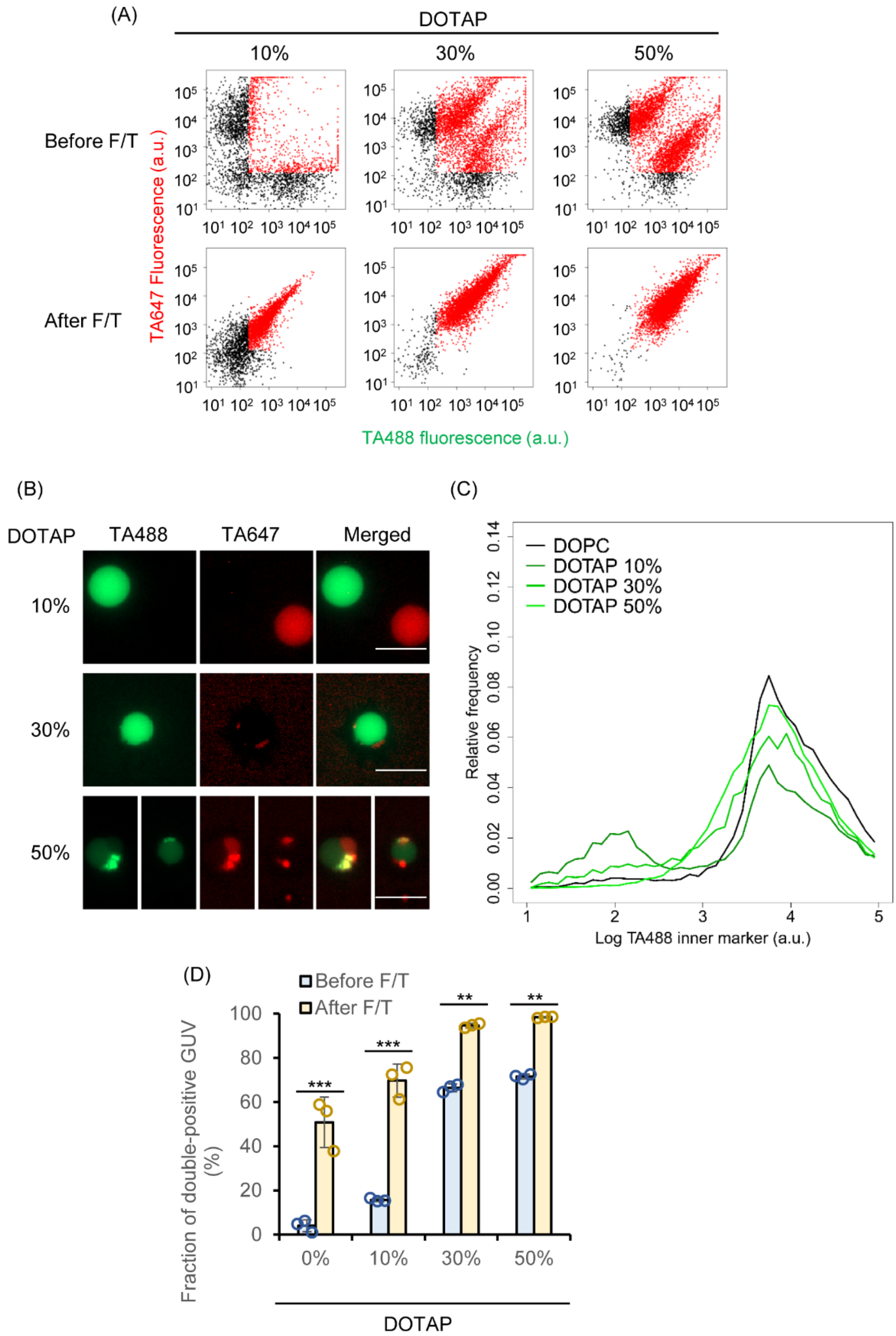

Supplementary Figure S4. Fluorescence analysis of encapsulated proteins in GUVs prepared from DOPC and DOTAP before and after freeze-thaw treatment. (A) Fluorescence of TA488 and TA647 measured by flow cytometry. Red dots indicate liposomes exhibiting fluorescence intensities of both TA488 and TA647 above the background level. (B) Histogram of TA488 fluorescence intensity inside GUVs before freeze-

thaw treatment at various DOTAP concentrations. The vertical axis represents the relative frequency. (C) Fluorescence microscopy images of liposomes containing TA488 (green) or TA647 (red) before freeze-thaw treatment. Scale bar, 10 μm. (D) Average fraction of double-positive liposomes within the gated GUV population, calculated as the number of double-positive GUVs divided by the total number of gated GUVs. Error bars represent the SD of three independent experiments ($n = 3$). Individual data points corresponding to each independent experiment are overlaid on the bar graphs. Statistical significance for comparisons between before and after F/T treatment is indicated as $^{**}p < 0.01$ and $^{***}p < 0.001$, based on Tukey's post hoc test.

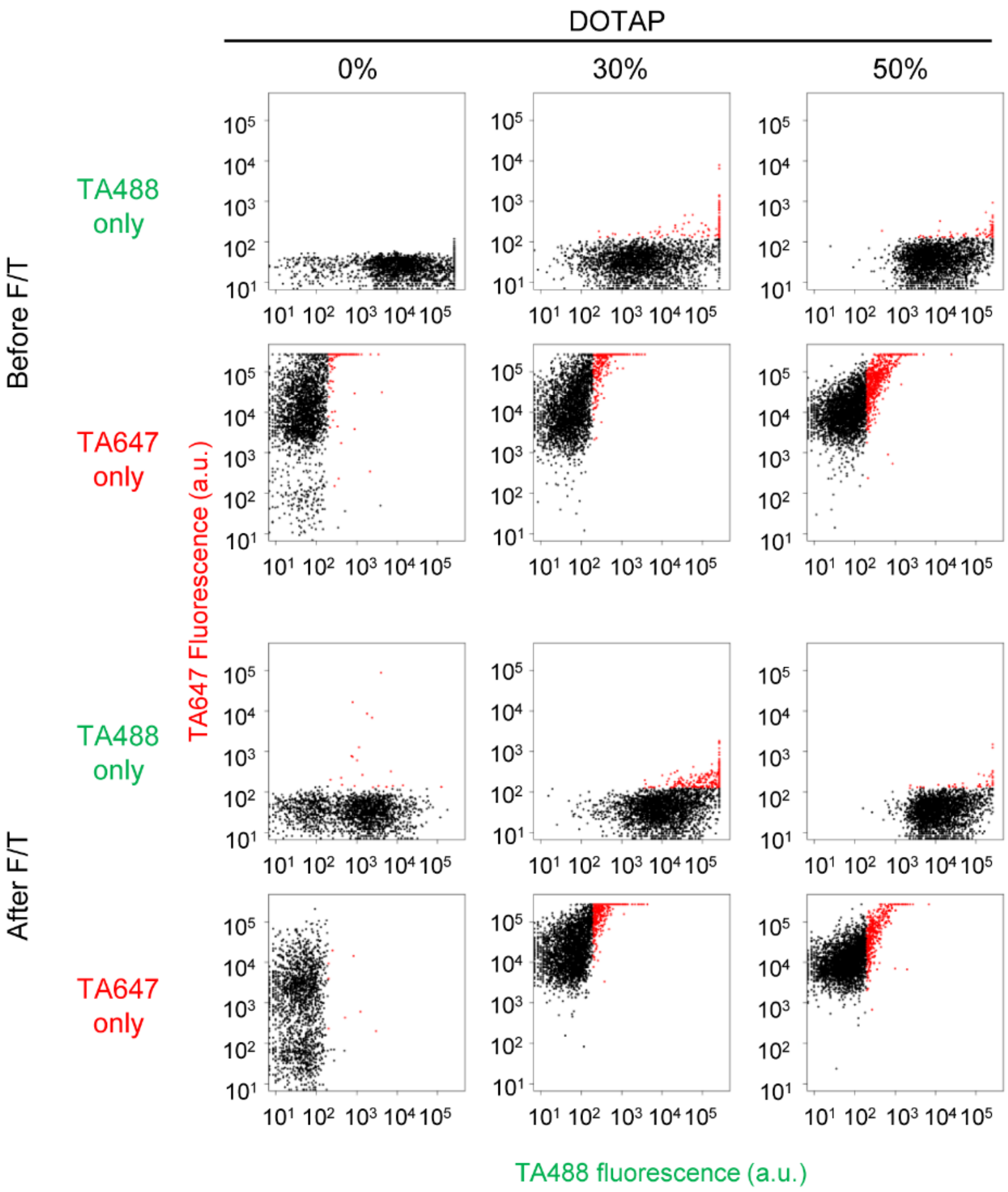

Supplementary Figure S5. Flow cytometry analysis of single-labeled liposomes before and after freeze-thaw (F/T) treatment. Fluorescence of TA488 and TA647 measured by flow cytometry. Red dots indicate liposomes exhibiting fluorescence intensities of both TA488 and TA647 above the background level.

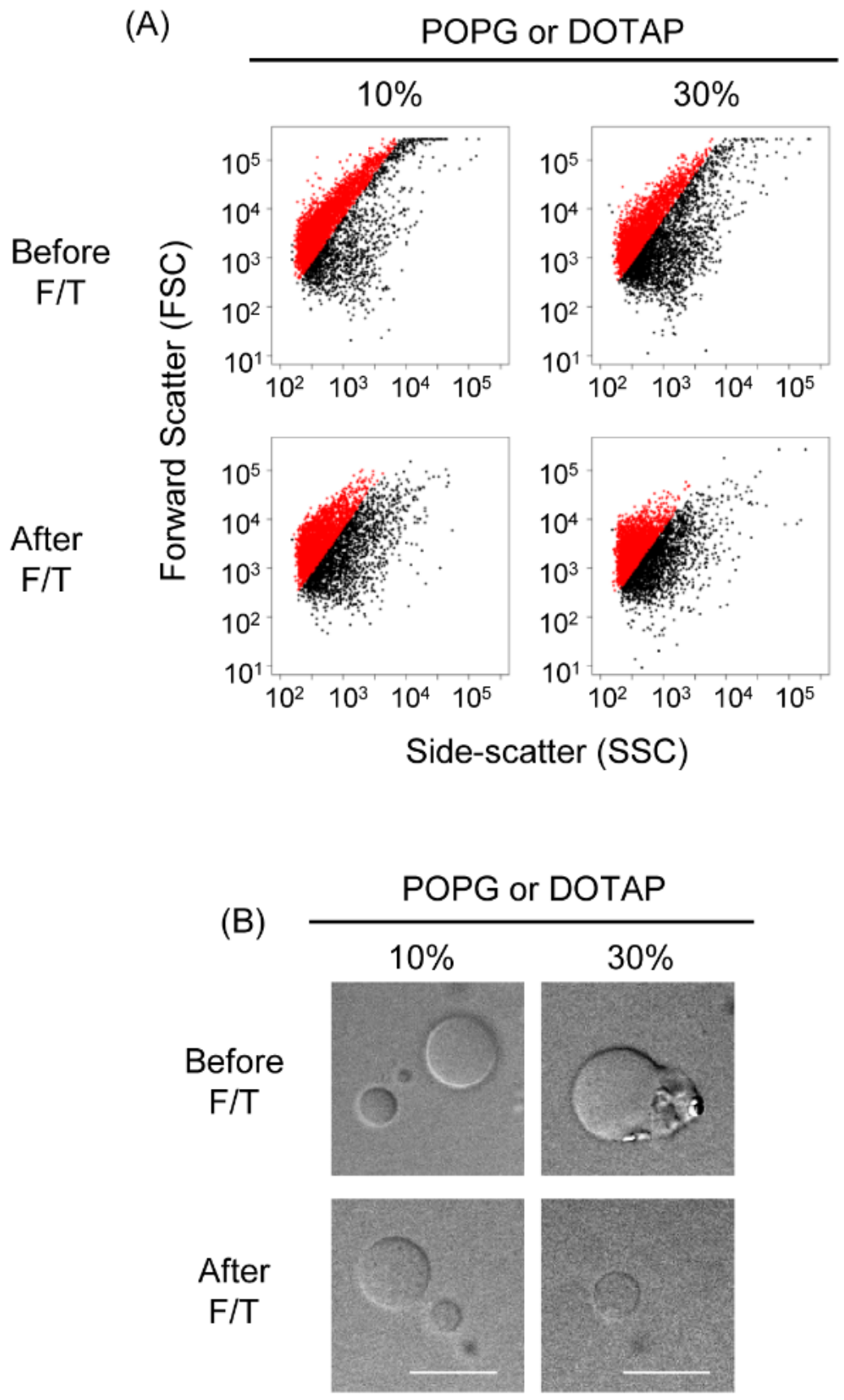

Supplementary Figure S6. Light-scattering and morphological analysis of mixed DOPG-containing GUVs and DOTAP-containing GUVs before and after freeze-thaw treatment. (A) Light-scattering analysis of liposomes measured by flow cytometry. Red dots indicate GUVs. (B) Differential interference contrast microscopy images of liposomes composed of DOPG- and DOTAP-containing GUV mixtures before and after freeze-thaw treatment. Scale bars, 10 µm.

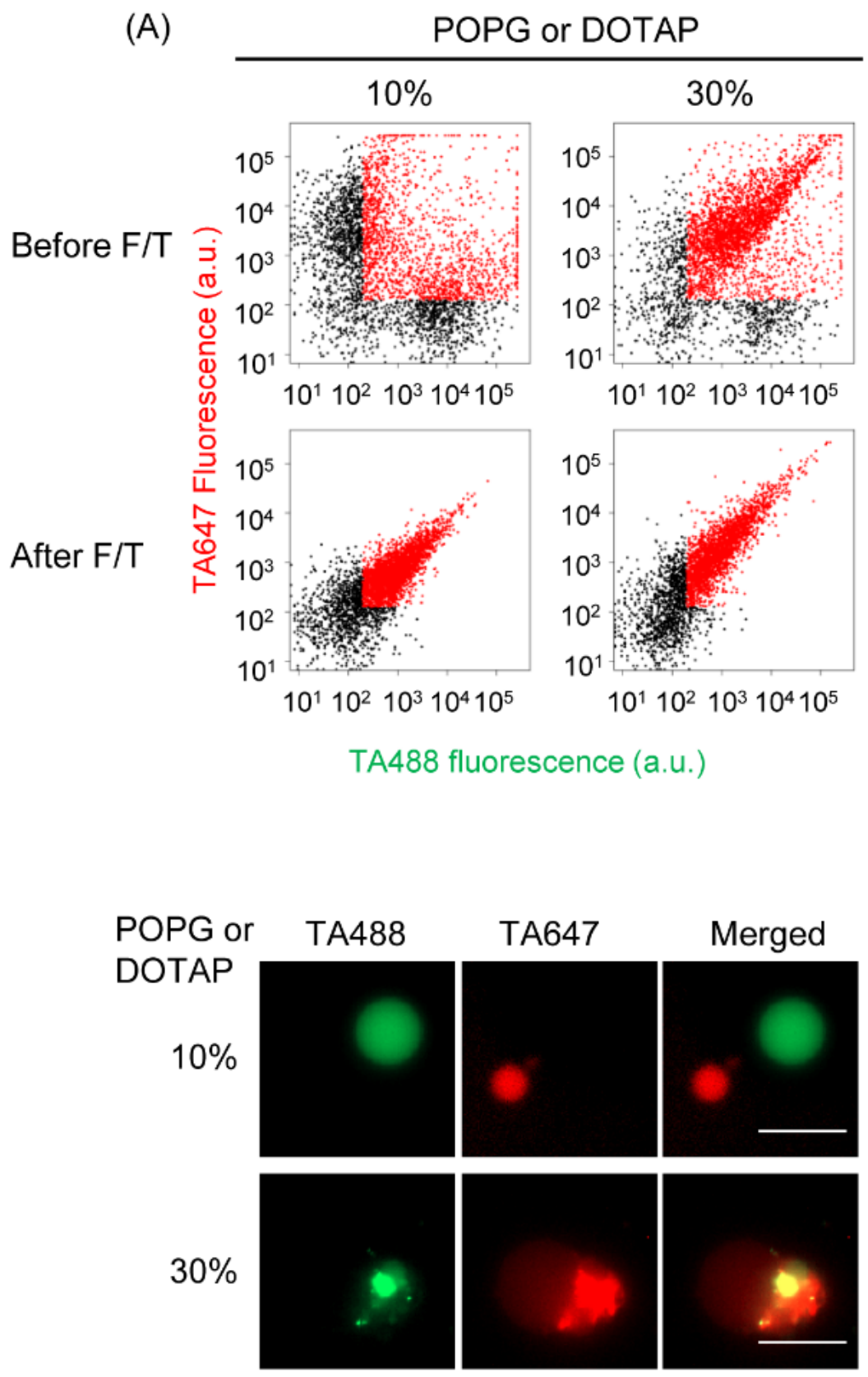

Supplementary Figure S7. Fluorescence analysis of encapsulated proteins in mixed DOPG-containing GUVs and DOTAP-containing GUVs before and after freeze–thaw treatment. (A) Fluorescence of TA488 and TA647 measured by flow cytometry. Red dots indicate liposomes exhibiting fluorescence intensities of both TA488 and TA647 above the background level, before and after freeze–thaw treatment. (B) Fluorescence microscopy images of liposomes containing TA488 (green) or TA647 (red) before and after freeze–thaw treatment. Scale bar, 10 µm.
